# The Humanitarian and Human Rights Resource Center: support to address global forensic issues

**DOI:** 10.1080/20961790.2017.1329055

**Published:** 2017-06-21

**Authors:** Douglas H. Ubelaker

**Affiliations:** Department of Anthropology, National Museum of Natural History, Smithsonian Institution, Washington, DC, USA

Dear Editor,

Global applications of forensic science are influenced by many factors. Notable among these are financial resources, training, equipment, maintenance of equipment, available professional expertise, political climate and administrative priorities [1]. Frequently, resources for forensic science applications are generated by governmental institutions and funnelled through their inherent bureaucracies. These applications focus on investigations related to suspected criminal activity but can also include more general humanitarian and human rights issues such as recovery and identification of decedents following mass disasters and political violence, as well as investigations of possible torture and abuse in custody. Unfortunately, global areas of greatest need for forensic resources also frequently are those with the least forensic capability and resources [2]. These issues have been addressed to some extent by active and responsive non-governmental organizations such as the Argentine Forensic Anthropology Team (EAFF), the Guatemalan Forensic Anthropology Foundation, Physicians for Human Rights, the International Commission on Missing Persons, the UK-based Inforce Foundation and the International Committee of the Red Cross [3–5]. The nature of the contributions made by these organizations and others reflects their individual charters and goals, as well as available resources.

## The American Academy of Forensic Sciences (AAFS)

While forensic scientists involved in the efforts listed above usually belong to key scientific organizations, their involvement primarily reflects their individual initiatives. In the past, many participants and even leaders of efforts to direct forensic science resources toward global areas of need have been members of the American Academy of Forensic Sciences (AAFS). In 2017, membership in the AAFS numbered about 6 600 representing forensic scientists from 72 countries. Founded in 1948, the AAFS is organized into 11 sections: Criminalistics, Digital and Multimedia Sciences, Engineering Sciences, Pathology/Biology, Questioned Documents, Jurisprudence, Anthropology, Toxicology, Odontology, Psychiatry and Behavioral Sciences and a General section. This Academy meets annually and provides those attending an active and dynamic forum to present research results and discuss key issues.

## The Humanitarian and Human Rights Resource Center (HHRRC)

In 2015, the AAFS Board of Directors recognized that while historically many of its members have been involved and provided leadership for the application of forensic science to global humanitarian and human rights problems, the organization has lacked formal recognition of those efforts, as well as any program specifically targeting those applications. To remedy this deficiency, the AAFS in 2015 formed the Humanitarian and Human Rights Resource Center (HHRRC). The concept of the new Center was developed by AAFS members attending the 2014 meeting of the International Association of Forensic Sciences and World Forensic Festival in Seoul, Korea. In particular, Luis Fondebrider of the EAFF, 2014–2015 AAFS President Daniel Martell and 2011–2012 AAFS President Douglas H. Ubelaker met in Seoul to suggest structure and purpose that was later augmented and approved by the AAFS Board of Directors in 2015. The title of the new program recognizes important differences between humanitarian and human rights applications, as well as the desired content as a “resource” for those involved in these types of applications.

As noted on their website (aafs.org) the AAFS Humanitarian and Human Rights Resource Center seeks to utilize the assets of the AAFS to promote the application of contemporary forensic science and forensic medicine principles to global humanitarian and/or human rights projects requiring special forensic assistance. The HHRRC also provides support to AAFS members engaged in human rights and/or humanitarian forensic applications and encourages AAFS members to increase their involvement in such matters. In addition to the assistance provided, the program recognizes that AAFS members are likely to develop their own personal and professional skills in undertaking this work. Discussions developing the statement of purpose language summarized above recognized that experienced colleagues engaging in such global projects usually learn more than they teach, emphasizing the personally enriching experience of such involvement.

In recognition of the “resource” component of the new HHRRC, subcommittees were formed to assemble and make available documents and publications, laboratory and analysis protocols, and educational materials. An additional subcommittee was assembled to evaluate equipment needs for recognized projects and to explore how these needs could be met at reduced cost. The Center established a database of volunteers from all sections of the AAFS who expressed a desire to become more extensively involved. In 2017, Chairs of the subcommittees were Marilyn Huestis of Toxicology (Publications and Documents), Sabra Botch-Jones of Toxicology (Laboratory and Analysis Protocols), Dawn Mulhern of Anthropology (Education) and Ron Singer of Criminalistics (Equipment).

To evaluate proposals for support, an International Advisory Council (IAC) was created. Members of the IAC in 2017 consist of Soren Blau (Australia), Stephen Cordner (Australia), Luis Fondebrider (Argentina), Michael Pollanen (Canada), Dawnie Steadman (USA), Morris Tidball-Binz (France), Duarte Nuno Vieira (Portugal), Oran Finegan (Switzerland) and Chair Douglas H. Ubelaker (USA). Proposals for support can include, but are not limited to, assistance with training, research applied to humanitarian and/or human rights projects, training materials and equipment, advisory services, as well as exchange of expertise, including that provided by AAFS members. Proposals are encouraged that are led and generated from within the country to which the proposal relates. Support for travel and per diem expenses, where required, is not to exceed the amounts recognized by the United States government for the cities and countries involved. No requests for salaries are considered, reflecting the humanitarian, volunteer spirit of this initiative. The HHRRC also does not support individual fellowships to study abroad or costs of routine DNA analysis. The amount of funding per individual project is limited to $20 000 US or less. The funding sources are directly from AAFS and from the National Institute of Justice, Forensic Technology Center of Excellence program administered by RTI International. Additional information about the HHRRC and how to apply for support is available on the AAFS website aafs.org. Requests for information about publications, documents, laboratory and analysis protocols and educational materials should be directed to the Chair of the HHRRC or the relevant subcommittee Chair. Information is also available on the website regarding how to make personal donations to the HHRRC.

In its first year, the HHRRC funded the following projects:

(1) Preserving evidence of the Khmer Rouge genocide: analysis and conservation of human skeletal remains in Cambodia and training of staff.
(2) Building forensic capacity in forensic archaeology and anthropology to help in the identification of human remains, with the participation of the families of the disappeared persons in Coahuila, Mexico.
(3) Application of stable isotope forensics to the identification of unidentified border crossers from the Texas-Mexico border.
(4) Strengthening (training) in the human identification Department of the PGJE (Procuraduria General de Justicia del Estado) in Tlaxcala, Mexico.
(5) A fully computerized method of osteometric sorting for pairwise comparisons in large assemblages (human remains).
(6) Detection of nerve agent exposure from human bone tissue.
(7) Technical assistance in establishing a forensic laboratory within the commission on human rights of the Philippines dedicated to the investigation of human rights violations.

Funds in 2017 are earmarked to support the following projects:

(1) Strategies for the identification of the 800 victims of the migrant shipwreck of April 18 2015 (Italy).
(2) Operation identification: exhuming the unidentified (Texas, USA).
(3) Detecting mass graves: broadening the knowledge base (Australia).
(4) Scene documentation for human rights investigators.
(5) Forensic investigations in the northern region of Guatemala.

Proposals are strongly encouraged from areas of the world and forensic units/projects of greatest need. If necessary, assistance is available from the HHRRC for language issues and general advice regarding proposal format and preparation.

## The Clyde Snow Award

Many long-time workers in forensic applications to humanitarian and human rights issues do not receive appropriate recognition for their tireless efforts. To partially remedy this situation, the HHRRC has created the Clyde Snow Award. This award seeks to recognize outstanding long-term accomplishments in the area of human rights and/or humanitarian forensic applications. The award is named for the late Clyde Snow (1928–2014) who pioneered forensic applications to such issues, especially in Latin America in the field of anthropology [3]. Nominations of individuals or organizations for this award should be submitted to the Chair of the HHRRC with a current copy of the nominee's curriculum vitae or the organization's founding documents and any relevant supporting documentation. The Clyde Snow Award will be presented by the Chair of the HHRRC at the Annual Business Meeting of the AAFS.

## Conclusion

In many areas of the contemporary world, problems emerge related to humanitarian issues and possible violations of human rights. Modern forensic science can contribute in many positive ways to the resolution of these issues. In some areas, these needs fall within the organizational and political cracks of normal institutional assistance and resolution. Such cases cry out for assistance from non-governmental organizations and individual volunteerism. The relatively new Humanitarian and Human Rights Resource Center of the AAFS joins these efforts in a positive attempt to make a difference.
